# ‘See what you feel’: The impact of visual scale distance in haptic-to-visual crossmodal matching

**DOI:** 10.1177/20416695251318591

**Published:** 2025-02-16

**Authors:** Olga Daneyko, Francesca Frisco, Angelo Maravita, Daniele Zavagno

**Affiliations:** Department of Psychology, Sociology and Politics, 7314 Sheffield Hallam University, Sheffield, UK; Department of Psychology, 9305 University of Milano-Bicocca, Milano, Italy; Milan Ceneter for Neuroscience, University of Milano-Bicocca, Milano, Italy

**Keywords:** haptic perception, crossmodal matching, haptic size perception, visual size perception, size constancy

## Abstract

Two experiments were conducted to explore the impact of the distance of a visual scale employed in the crossmodal matching method dubbed *See What You Feel* (SWYF) used to study the Uznadze haptic aftereffect. Previous studies reported that SWYF leads to a general underestimation of out-of-sight handheld spheres, which seems to increase with visual scale distance. Experiment 1 tested the effect of visual scale distance in haptic-to-visual crossmodal matching. A 19-step visual scale, made of actual 3D spheres (diameters ranging from 2.0 to 5.6 cm), was set at one of three possible distances (30, 160, 290 cm); participants’ task was to find the matching visual spheres for four out-of-sight handheld test spheres (diameters 3.0, 3.8, 4.6, 5.0 cm). Results confirmed the underestimation effect and only partially confirmed the role of scale distance. Experiment 2 investigated the role of scale distance in a visual-to-visual matching task in which the same visual scale was employed, set at one of three distances (37, 160, 290 cm). Participants’ task was to find a match for the same four test stimuli. Results showed no statistical difference between matched and actual sphere sizes with distance 37 cm; underestimations were observed with the far distances, thus reflecting overestimations of scale sphere sizes. Results from both experiments allow us to conclude that the underestimation effect observed with SWYF is a general feature of haptic-to-visual crossmodal matching, and that the SWYF method is a valuable tool for measuring haptic size perception with handheld stimuli when the visual scale is set at a visually comfortable peripersonal distance.

## How to cite this article

Daneyko, O., Frisco, F., Maravita, A., Zavagno, D. (2025). ‘See what you feel’: The Impact of visual scale distance in haptic–to–visual crossmodal matching. *i–Perception*, 16(0), 1–17. https://doi.org/10.1177/20416695251318591

The purpose of this study is to investigate the impact of viewing distance on the precision and accuracy of haptic size estimations obtained through a visual matching task within the context of crossmodal psychophysical paradigms. This research builds on previous work (Daneyko et al., 2020; Frisco et al., 2023) aimed at measuring and studying the Uznadze haptic aftereffect^1^ by employing a crossmodal matching paradigm dubbed ‘See What You Feel’ (SWYF). In the SWYF experiments, participants were asked to find a match for the spheres they were grasping out of sight on a three-dimensional visual scale made of spheres organised from the smallest (left side) to the biggest (right side). The method was designed to provide an easily understandable matching task (Zwislocki, 1991) to measure the haptic illusion. The approach aimed at avoiding potential ‘contamination’ of results that might arise from the adaptation process necessary to induce the illusion, a situation that could occur if a haptic measurement task were to be involved.^2^

While in our previous studies we focused on the Uznadze illusion, in this study we expand our research by moving away from the illusion to consider the SWYF method in itself, to explore a factor that can affect in a significant way matching results, that is, the distance between the observer and the 3D visual scale.

Crossmodal matching is a paradigm often used to explore vision and haptic interactions (Klatzky et al., 1985; Norman et al., 2012; Pinardi et al., 2023; Rock & Victor, 1964). The SWYF method may appear similar to several approaches that used haptic-to-visual crossmodal matching (e.g., Brenner & van Damme 1999*;* Héroux et al., 2024; Van Doom & Symmons, 2013). However, it involves important differences: (a) comparison stimuli are actually three dimensional, not pictorial; (b) they are organised as a horizontal scale with a specific orientation, from small on the left to large on the right, thus mimicking reflectance scales widely employed to study lightness perception (Zavagno et al., 2010); (c) and scale distance is an independent variable, which has not been considered in haptic-to-vision research that employed a matching method.

The structured approach of measuring leverages the advantages of scales, which not only provide an easily approachable and systematic way to measure perception but also enhance effectiveness and may reduce bias with respect to other psychophysical methods. Empirical evidence from Daneyko et al.'s study supports the validity of the method, showing results that are consistent with findings reported in previous studies on the direction of the Uznadze haptic aftereffect (Kappers & Bergmann Tiest, 2013; Khachapuridze, 1962; Maravita, 1997; Piaget & Lambercier, 1944) while employing a method that allows to assess the size of the illusion through an easy and cognitively nondemanding task.

Nevertheless, it is important to recognise that no method can claim complete validity in all circumstances. For instance, when visually assessing perceived size, the obtained estimations can be affected by various factors, including viewing distance (Gilinsky, 1951; Carlson, 1960), a factor commonly ignored in crossmodal studies on haptics and vision. Daneyko et al. (2020) observed that the distance of the visual matching scale in the SWYF method can indeed impact on matching results. In general, with a visual scale placed at 30 cm from participants, that is, within peripersonal distance (space within hand reach), it was found that the size of handheld test spheres was underestimated with respect to their actual size in the visual scale. A similar underestimation effect was recently observed by Frisco et al. (2023) who employed the SWYF method to study the effect of arm posture on the Uznadze haptic aftereffect. However, Daneyko et al. found underestimations to be bigger when the visual scale was placed at 160 cm from the participant, that is at an ‘out of reach’ distance, commonly defined as extrapersonal space. Hence, to validate, it is imperative to understand the role played by the distance between participants and the visual scale.

Previous research into size-judgement reported a trend for underestimating the size of visual stimuli at distances of 6 m (Kavšek & Granrud, 2012). There is, instead, a pattern of overestimation in the size of visual stimuli when stimuli are observed from far distances, for instance from 61 m (Carlson, 1962; Epstein, 1963; Gilinsky, 1955; Kavšek & Granrud, 2012). One possible reason for the overestimation effect could be the strategic inflation of size estimates. Because the precise degree of this hypothesised inflation remains uncertain, it has been suggested that participants may rely on heuristics when making guesses, which can often result in overestimating the actual size of an object (Granrud, 2012; Teghtsoonian, 1974; Wohlwill, 1963). No comments or explanations have been provided regarding the underestimation effect observed at closer distances, such as 6 m.

As far as our knowledge goes, currently there is no available research specifically conducted on visual size estimation at shorter distances, such as 30 and 160 cm. In addition, previous research has mainly used only one sensory modality for size processing. Specifically, early studies typically involved participants comparing the size of visual stimuli at different distances to a reference stimulus, which was also visual (Carlson, 1962; Epstein, 1963; Gilinsky, 1955; Kavšek & Granrud, 2012).

Regarding haptic perception, earlier studies (Katz, 1925; Gibson, 1963; Klatzky et al., 1985) have suggested that participants can identify both familiar and unfamiliar objects using touch alone. However, subsequent investigations have unveiled that when it comes to specific properties such as shape, curvature (Kappers, 2011), and size (Craddock & Lawson, 2009), haptic perception is subject to certain limitations. This suggests that participant's ability to discern objects through touch could vary significantly depending on the characteristics and complexity of the objects.

In contrast, the SWYF method combines information from both the visual and haptic modalities. These methodological differences may potentially contribute to the observed underestimation effect at shorter distances reported by Daneyko et al. (2020) and Frisco et al. (2023). In fact, when considering the interaction between vision and touch, existing literature demonstrates that both senses provide valuable information about specific object properties such as size, texture, and shape (Ernst & Banks, 2002; Ernst et al., 2000; Gepshtein & Banks, 2003; Lederman & Abbott, 1981; Norman et al., 2012; Rock & Victor, 1964). However, in cases of inconsistency between the information received from vision and touch, vision tends to dominate, likely due to the perception of visual information as being more accurate and informative (Ernst & Banks, 2002; Helbig & Ernst, 2007; Lederman & Klatzky, 1997).

The utilisation of a visual scale made of actual three-dimensional objects is relatively new in crossmodal haptic-to visual matching and it has only been used to study the Uznadze illusion. Therefore, it is important to test its limitations, particularly in relation to the distance at which the visual matching scale is placed with respect to the perceiver. This study extends our previous work by focusing on the following areas:

1. Assessing whether the SWYF method could be applied in non-illusory contexts, thereby validating its general utility as a crossmodal matching method.
2. Investigating whether the underestimation effect observed in our previous work is generally present in non-illusory haptic-to-visual matching;
3. Determining whether the underestimation effect is a characteristic trade-off of the SWYF method resulting from the interaction between visual and haptic perception.
4. Exploring how the distance between participants and the visual scale affects measurement accuracy and consistency.

Therefore, in experiment 1 we tested the impact of three different visual scale distances on the estimation of the size of spheres handheld out of sight. In experiment 2 we explored how visual-to-visual matching is affected in our experimental setup, by employing the same visual scale and its distances from participants. The primary aim of experiment 2 was to isolate visual matching from haptic input to verify if the observed underestimation effect is specifically related to a crossmodal interaction.

## Experiment 1: The Influence of Scale Distance on Haptic Size Estimation Through Visual Matching

### Participants

Forty-five participants (12 males, mean age 23) took part in the experiment. Participants were students and postdocs from the Psychology Department at the University of Milano-Bicocca. All participants were righthanded, as determined with the Edinburgh Inventory (Oldfield, 1971). All participants had normal or corrected-to-normal vision and were initially unaware of the experiment's purpose. The experimental protocol was explained to all participants who gave written informed consent to participation. The experiment was approved by the Research Evaluation Committee of the Department of Psychology.

### Experimental Design

This study follows a mixed design, with *sphere* (diameters: 30, 38, 46 and 50 mm) and *hand* (the hand grasping a sphere: left, right) as within-subjects factors, and *distance* (scale distance from the observer: 30 cm, 160 cm and 290 cm) as between-subjects factor.

The 45 participants were evenly and randomly distributed among the three *distance* conditions. Visual matches were the dependent variable. Specifically, participants were required to select a sphere on the visual scale that matched in size the sphere they were clenching out of sight in their hand.

### Materials

**Figure 1.**
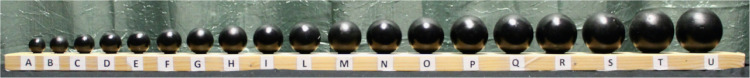
The visual scale: 3D-printed spheres, ranging from 20 mm to 56 mm in diameter and labelled from A to U (from left to right), placed in sequence on a wooden rod.

The visual scale consisted of 19 spheres created using a 3D printer. The spheres were organised in ascending order from left to right and attached to a specifically designed wooden rod measuring 87 cm in length (Figure 1). The diameter of the smallest sphere on the scale measured 20 mm, the diameter of the biggest measured 56 mm; the difference in size between neighbouring spheres on the scale was 2 mm and each subsequent sphere increased in diameter by 2 mm. The wooden rod showed a label under each sphere, with the smallest sphere labelled as *A* and the biggest as *U*. The scale was positioned at three different distances: 30 cm, 160 cm, and 290 cm from the participant, always at the same height from the floor (91 cm), roughly at participants’ eye level, who were seated on a chair adjustable in height. From those three distances, visual angles of the smallest scale sphere (20 mm) measured approximately 3.81, 0.71 and 0.39°; visual angles of the biggest scale sphere (56 mm) measured approximately 10.57, 2.00 and 1.10°.

Four handheld test spheres (TS) were employed, which measured 30, 38, 46 and 50 mm in diameter, corresponding in size to the scale spheres labelled F, L, P and R. These sizes were chosen to maximise differences between test stimuli while remaining well within the matchable range of the visual scale. Hence, test sphere 50, R on the scale, was chosen instead of a test sphere measuring 54 mm (corresponding to T on the visual scale) because in case of overestimations a match might have been impossible to find. The test spheres were created using the 3D printer; they were hollow and could be taken apart by unscrewing the two halves. This enabled the three smaller spheres to be filled with small fishing sinkers and cotton ensuring that, regardless of its size, each sphere was standardised to weigh the same as the largest test sphere (28 g).

### Procedure

The experiment took place in a narrow windowless rectangular laboratory in which walls and ceiling are matte black. The room was normally illuminated from above. The visual scale, made of black spheres, was positioned on a black table and in front of a white panel that served as background to maximise its visibility. The three different visual scale distances were achieved by moving the table to specific marks on the floor.

Participants were seated at a black working table with an underneath space open on both sides, allowing them to insert their arms through the aperture of the table and rest them comfortably. This setup ensured that participants could not see the handheld spheres, facilitating the researcher's task of placing these out of sight into their hand. It also ensured a constant distance between participants and the visual scale within each distance group. Once a sphere was positioned in the participants’ hand, they were instructed to clench the latter and feel its size. Then, they were asked to select a sphere from the visual scale – positioned in front of them at one of the three distances – that matched in size the sphere they were holding. This process was repeated for each of the four test spheres presented in random order. Participants first evaluated all four spheres with their left hand and then with their right hand. This sequence was repeated other two times; the total number of trials was thus 24. The participants had no time restrictions but were encouraged to complete the matching task promptly.

After completing the experiment, a brief explanation regarding the purpose of the study was provided. On average, the duration of the experiment was approximately 20 minutes.

### Results and Discussion

For each participant, mean matchings were calculated for the test spheres in relation to each hand. Hence, the actual diameter of a sphere was subtracted from the diameter of its mean match, which represented the perceived difference in size between the haptically held sphere in a specific hand and its actual size; for instance $\Delta 30L=\bar{M}30\;Left-30\;Actual$ , and $\Delta 30R=\bar{M}30\;R-30\;Actual$ . Thus, if a sphere is matched with a sphere bigger than its actual size, *Δ* would be positive; vice versa, if it is matched with a smaller sphere, *Δ* would be negative. *Δ* represents the independent variable we considered in our analyses, and it is expressed in mm.

Descriptive statistics were calculated to assess the normality of ΔL and ΔR data distributions for each test sphere in relation to scale *distance* (30, 160 and 290 cm, Table 1). Three potential outlier scores have been identified using the interquartile range (IQR) method for outlier detection (Aggarwal, 2015): one with distance 30 cm and two with distance 160 cm. However, even after removing these outlier scores, the statistical outcome remained unaffected. Considering the limited number of participants, it has been decided to retain the outlier scores in the dataset. Δs for factors *sphere*, *hand* and *distance* are normally distributed, except for Δ30L matched at the distance of 290 cm (Shapiro–Wilk test: *W *= 0.872, *p* = .036, M = −2.49, SD = 3.92 (95% CI −4.66, −0.32). Skewness and kurtosis values were both within the acceptable range of −1.5 to +1.5 for smaller sample sizes (e.g., 30–50 participants; Tabachnick & Fidell, 2013).

**Table 1.** Experiment 1: Descriptive statistics for sphere*distance.

Distance	Δ 30 mm			Δ 38 mm			Δ 46 mm			Δ 50 mm		
	30	160	290	30	160	290	30	160	290	30	160	290
**Mean**	−2.53	−4.29	−2.81	−2.40	−5.82	−4.11	−3.09	−7.82	−5.73	−2.74	−6.82	−4.92
**SD**	2.48	2.11	4.14	2.01	2.67	5.1	2.02	2.91	6.60	1.79	2.70	5.78
**95% C.I.**	**−**1.27	−3.21	−0.71	−1.38	−4.47	−1.50	−2.07	−6.35	−2.39	−1.83	−5.45	−1.99
	**−**3.79	−5.36	−4.91	−3.42	−7.17	−6.71	−4.11	−9.29	−9.07	−3.65	−8.19	−7.84

An ANOVA for repeated measures, with Greenhause-Geisser sphericity corrections, was thus calculated with *sphere* (30, 38, 46, 50 mm) and *hand* (left, right) as within- and *distance* (30; 160; 290 cm) as between-subjects factors. The analysis revealed significant main effects for all three factors: *sphere F*(1.920, 80.634) = 25.549, *p *< .001, *η*^2^_p _= .378; *hand F*(1, 42) = 7.353, *p *= .010, *η*^2^_p _= .149; *distance F*(2, 42) = 4.446, *p *< .018, *η*^2^_p _= .175. Only the interaction sphere*distance determined significant effects on the matching task: *F*(3.840, 80.634) = 4.952, *p* = .001, *η*^2^_p _= .191. None of the other interactions were significant (*p* values > .3). The main effect of *distance* is consistent with the findings reported by Daneyko et al. (2020), however, the main effect of *hand* was not reported by Daneyko et al. or by Frisco et al. (2023). With regards to *hand*, results show greater underestimation of sphere sizes for the right hand than for the left one (right hand marginal mean = −4.947, S.E. 0.554; left hand = −4.392; S.E. 0.554). It would be interesting to see if such results are mirrored by left-handed adults.

Given that *hand* did not interact with the other two factors and that it did not determine significant effects in the results reported by Daneyko et al. and Frisco et al., we may safely conclude that such factor does not play a significant role in SWYF crossmodal matching tasks with righthanded individuals. In the subsequent analyses and discussion, the factor *hand* will not be taken into further consideration, with data for the two hands averaged.

**Figure 2.**
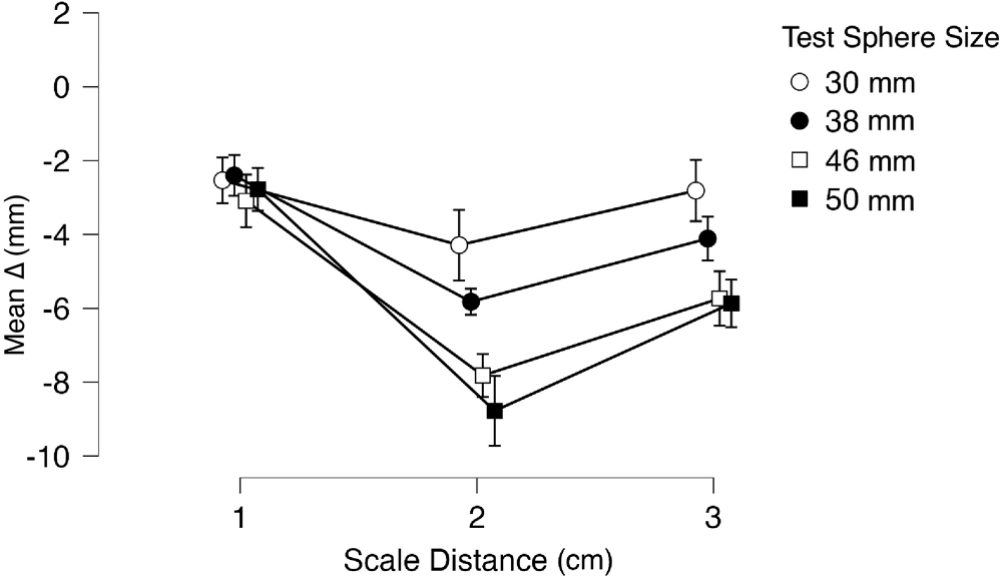
Experiment 1: mean *Δs* (see main text) for each test sphere distinguished by distance. The graph shows a general haptic size underestimation with visual matching. Error bars represent C.I. intervals.

Figure 2 shows mean *Δs* distinguished by scale distance for the four test spheres. The graph shows five interesting things: (a) all handheld test spheres were underestimated with respect to their actual scale size (all *Δs* < 0); (b) *Δs* for all four test spheres appear equivalent when the scale is placed at 30 cm from the observer, that is, within peripersonal space; (c) *Δs* are both greater and statistically different from each other with the scale distance set at 160 cm (extrapersonal space); (d) while still different in size, all *Δs* decrease when the scale distance is set at 290 cm; (e) such decrements are greater for the two smaller test spheres with scale distance set at 290 cm. With reference to point 1, one-sample *t*-Tests confirm that for all three distances *Δs* are statistically different from 0 (*p *< .001), while with regards to point 2, unpaired-samples *t*-Tests confirm that at scale distance 30 cm *Δs* from all four TS are statistically equivalent (*p *> .3). With reference to points 3 and 4, unpaired *t*-Tests were conducted to compare the *Δs* of a TS across the three scale distances (Table 2): for all test spheres, a statistical difference was found only between distances 30 and 160 cm.

**Table 2.** Experiment 1: Results for unpaired *t*-Tests (df=28) (see main text).

	*Δ* 30 mm		*Δ* 38 mm		*Δ* 46 mm		*Δ* 50 mm	
Distance	30 cm	160 cm	30 cm	160 cm	30 cm	160 cm	30 cm	160 cm
**160 cm**	*t* = 2.08	/	*t* = 3.96	/	*t* = 5.17	/	*t* = 4.86	/
	*p *= .046		*p *< .001		*p *< .001		*p *< . 001	
**290 cm**	*t* = .22	*t* = −1.22	*t* = 1.19	*t* = −1.43	*t* = 1.48	*t* = −1.12	*t* = .1.39	*t* = −1.15
	*p *> . 8	*p *> .2	*p *> .2	*p *> .2	*p *> .1	*p *> .2	*p *> .1	*p *> .2

**Figure 3.**
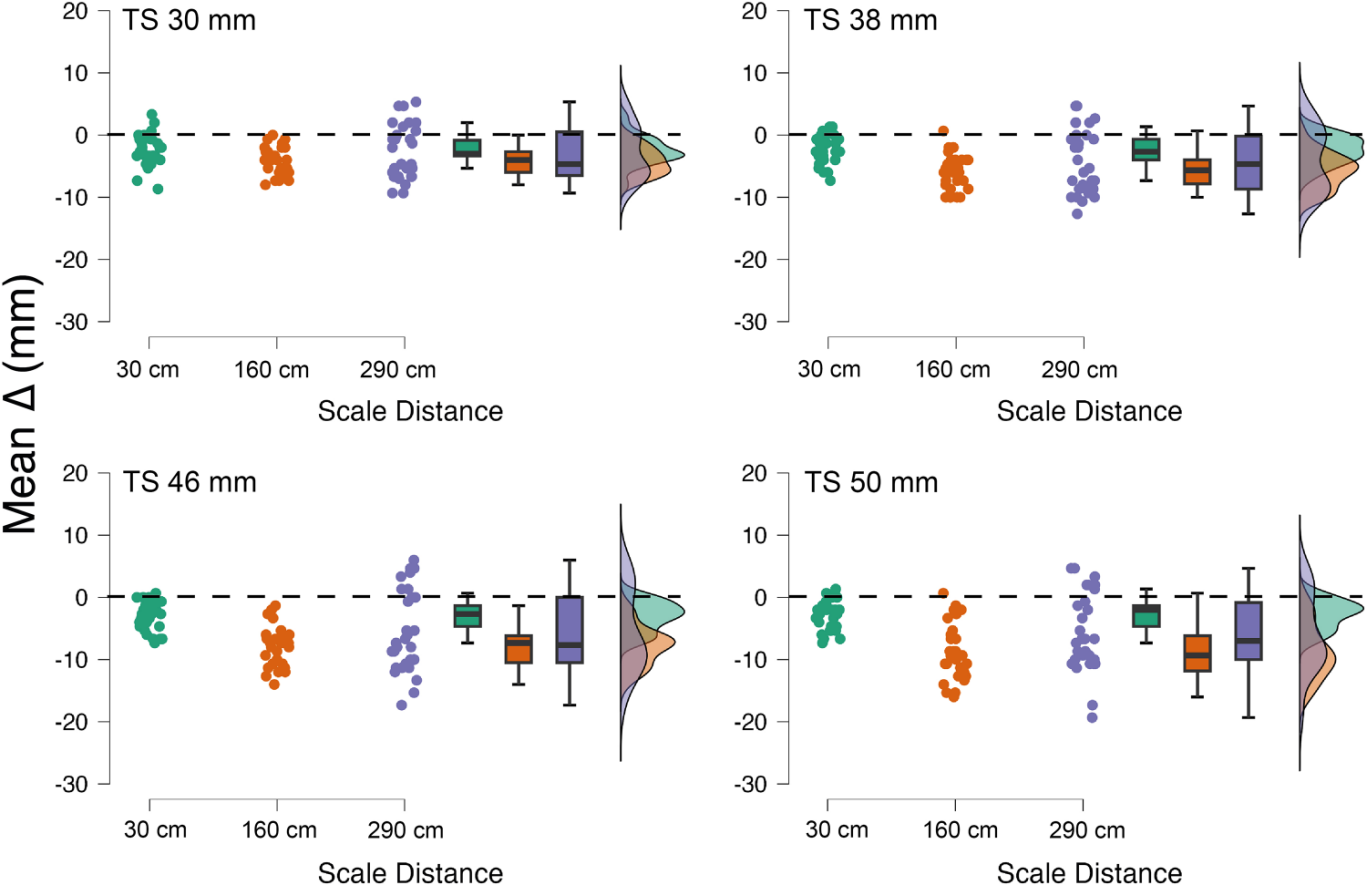
Experiment 1: data distributions for the four test spheres as a function of visual scale distance.

Points 3–5 are best explained by considering the graphs plotted in Figure 3 and the mean SD values for each TS matched at the three visual scale distances (Table 1). As shown in the plot, mean *Δs* vary depending on the distance condition, leading to V-shape patterns for all test spheres. Specifically, mean *Δs* tend to increase at 160 cm compared to 30 cm, indicating a greater underestimation of size with the increase in the visual scale distance. However, when the visual scale is set at 290 cm, mean *Δs* surprisingly decrease (i.e., less underestimation) for all spheres. Nevertheless, increments in SD are observed at 290 cm, indicating that as the distance of the visual scale increases, the task could become more challenging, leading to greater variability in participants’ responses. Thus, the reduced underestimation at 290 cm is accompanied by an increase in uncertainty and a reduction in the precision of participants’ estimations, resulting in a greater dispersion of matching results.

To sum up the results of Experiment 1, the analyses confirm an underestimation of the test spheres on the visual scale for all scale distances, which is consistent with results from Daneyko et al. (2020) and Frisco et al. (2023). Moreover, the entity of the underestimations is statistically equivalent among the four test spheres when the scale is placed at a distance of 30 cm from participants, that is, within peripersonal space. Such equivalence however vanishes as the sizes of the test spheres and the distance of the visual scale increase. Moreover, with a scale placed at 290 cm from participants, matches are largely dispersed along the visual scale.

The underestimation effect appears to be a constant feature of the SWYF method. But is it intrinsic to the method itself, involving the integration of haptic and visual information, or does it depend on a loss of size constancy related only to the distance of the visual scale? Moreover, we cannot determine whether this tendency reflects a general overestimation of the size of visual stimuli placed on the visual scale or whether it is due to a haptic underestimation of the manipulated stimulus size. To tackle these issues a second experiment was conducted in which participants were asked to find a match on the visual scale set at distances comparable to those employed in experiment 1, while test stimuli were not handheld but placed in front of them (visual to visual matching).

## Experiment 2: The Effect of Scale Distance on Visual-to Visual Matching

### Participants

Forty-five participants (18 males, mean age 25), all students from the University of Milano-Bicocca, and none of whom participated in experiment 1, took part in the experiment. All participants had normal or corrected-to-normal vision and were initially unaware of the experiment's purpose. The experimental protocol was explained to all participants who gave written informed consent to participation. Though irrelevant to the task, participants were all righthanded as determined with the Edinburgh Inventory (Oldfield, 1971). The experiment was approved by the Ethics Committee of the Department of Psychology.

### Materials and Procedure

The experimental design was very similar to Experiment 1, with one key difference: participants were required to perform a visual-to-visual match. Indeed, the four test spheres (30, 38, 46, 50 mm) were this time viewed, not touched, and participants were tasked with finding their match on the visual scale set at 37, 160 or 290 cm. Test spheres were placed on a table positioned close to the participant's location, viewed from 32 ± 1 cm. A scale distance of 37 cm was chosen over that of 30 cm employed in experiment 1 to allow enough viewing space between the test spheres and the visual scale, which was positioned on the black table. The visual angles of the smallest and the biggest spheres from such distance were respectively 3.09 and 8.60°. As in experiment 1, the distance between floor and the base of the scale was 91 cm, roughly at participants’ eye level. Test spheres were positioned in front of the observer 8 cm lower than the visual scale. Participants were randomly assigned to one of the three scale distances; while the matching scale remained always visible, test spheres were presented three times each, one at a time in semirandom order. Therefore, consistent with the approach used for each hand in Experiment 1, participants performed 3 matches for each sphere, for a total of 12 trials. Participants were instructed to sit with their backs straight, keeping their bodies stable between the chair back and the table.

### Results and Discussion

As in Experiment 1, for each participant we calculated the mean visual match for each test sphere. We then calculated *Δ*_v_ (v = visual) values as the difference between visual matches and actual sphere diameters on the scale: *Δ*_v _= *mean match – actual size*. Descriptive statistics were then calculated to assess the normality of *Δ*_v_ data distributions for each test sphere in relation to scale *distance* (37, 160 and 290 cm). Using the IQR method for outlier detection (Aggarwal, 2015), we identified four potential outliers: two with distance 160 cm and two with distance 290 cm. Nevertheless, upon removing these outliers the results align with those of the entire sample. As a result, the decision has been made to retain the complete sample.

The *Δ*_v_ data for both factors *sphere* and *distance* were normally distributed, except for one sphere (30 mm) matched from 37 and 290 cm, respectively: Shapiro–Wilk test: W = 0.802, *p* < .05 M = −4.44e−17, SD = 1.43 (95% CI −0.789, 0.789; W = 0.862, *p* < .05) M = −1.73, SD = 2.65 (95% CI −3.20, −0.26). The skewness and kurtosis values were generally within the acceptable range of −1.5 to +1.5 for smaller sample sizes. However, there was an exception in the case of sphere 30 cm at the distance of 37 cm, where the kurtosis value was 3.76 and the skewness was −1.82.

**Figure 4.**
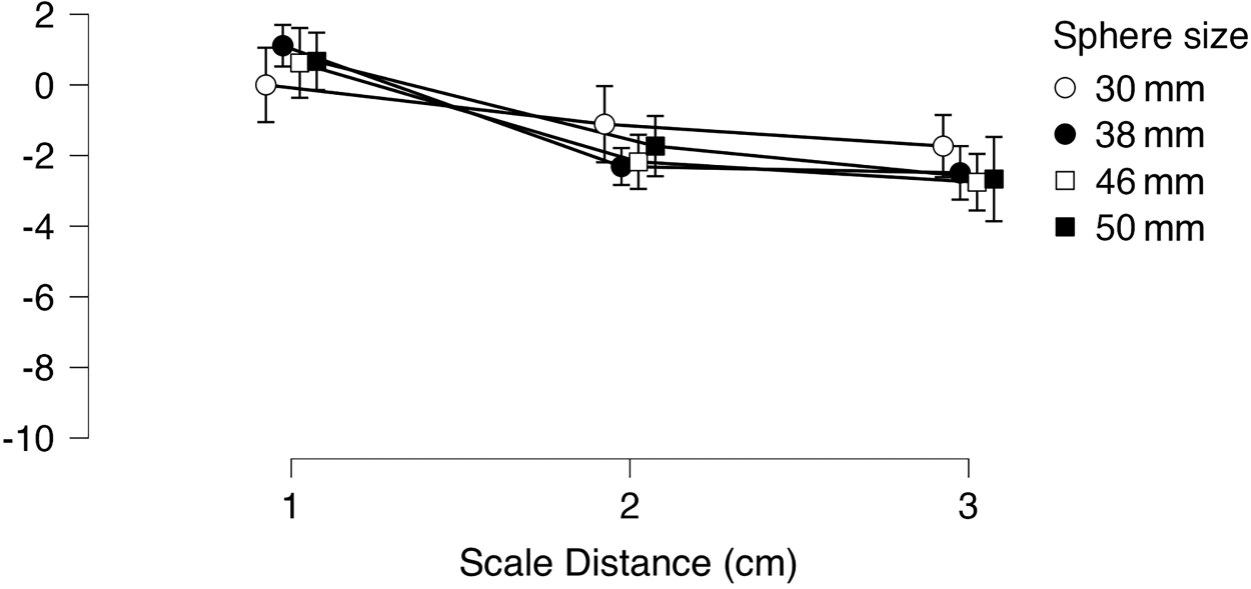
Experiment 2: mean *Δ*v for each test sphere distinguished by visual scale distance. Error bars represent confidence intervals.

Given that only the measurements of test sphere 30 cm at distances of 37 and 290 cm yielded non-normally distributed data, a repeated measures ANOVA was conducted on the dataset with *sphere* (30, 38, 46, 50 mm) as within and *distance* (37, 160, 290 cm) as between factors. The analysis shows a significant main effect solely for *distance*: *F*(2, 42) = 7.60, *p *= .002, *η*_p_² = .266. Neither the main effect of *size* (*p = .*539) nor the interaction sphere*distance (*p *= .109) were statistically significant. Post hoc comparisons with Bonferroni correction indicated that test spheres *Δ* with scale distance set at 30 cm are significantly different from those with the scale set at 160 cm (*t*(42) = 2.97, *p *= .015) and 290 cm (*t*(42) = 3.67, *p *= .002); instead, measurements carried out with the scale set at 160 and 290 cm are not statistically different (*p *= 1). Table 3 reports descriptive statistics for Experiment 2; Figure 4 shows mean *Δ*_v_ values for each test sphere distinguished by visual scale *distance*. One sample *t*-Tests were carried out on *Δ*_v_s relative to *distance* 37 cm to verify whether mean matches are statistically different from the actual scale sizes of the spheres (0 on the *y*-axis in Figure 4). Only the test sphere measuring 38 mm is statistically different from its actual scale size: *t*(14) = 3.849, *p *< .005, d = .99.

**Table 3.** Experiment 2: Descriptive statistics for sphere*distance.

Distance cm	Δv 30 mm			Δv 38 mm			Δv 46 mm			Δv 50 mm		
	37	160	290	37	160	290	37	160	290	37	160	290
**Mean**	2.227 × 10^−17^	−1.11	−1.73	1.11	−2.31	−2.49	0.62	−2.17	−2.75	0.66	−1.73	−2.66
**SD**	1.42	2.56	2.65	1.11	2.37	2.71	2.47	3.04	3.20	2.03	3.10	3.70
**95% C.I.**	0.72	−3.21	−0.39	1.67	−1.10	−1.11	1.87	−0.63	−1.13	1.69	−0.16	−0.79
	**−**0.72	−5.36	−3.07	0.54	−3.51	−3.86	−0.63	−3.72	−4.37	−0.36	−3.30	−4.54

**Figure 5.**
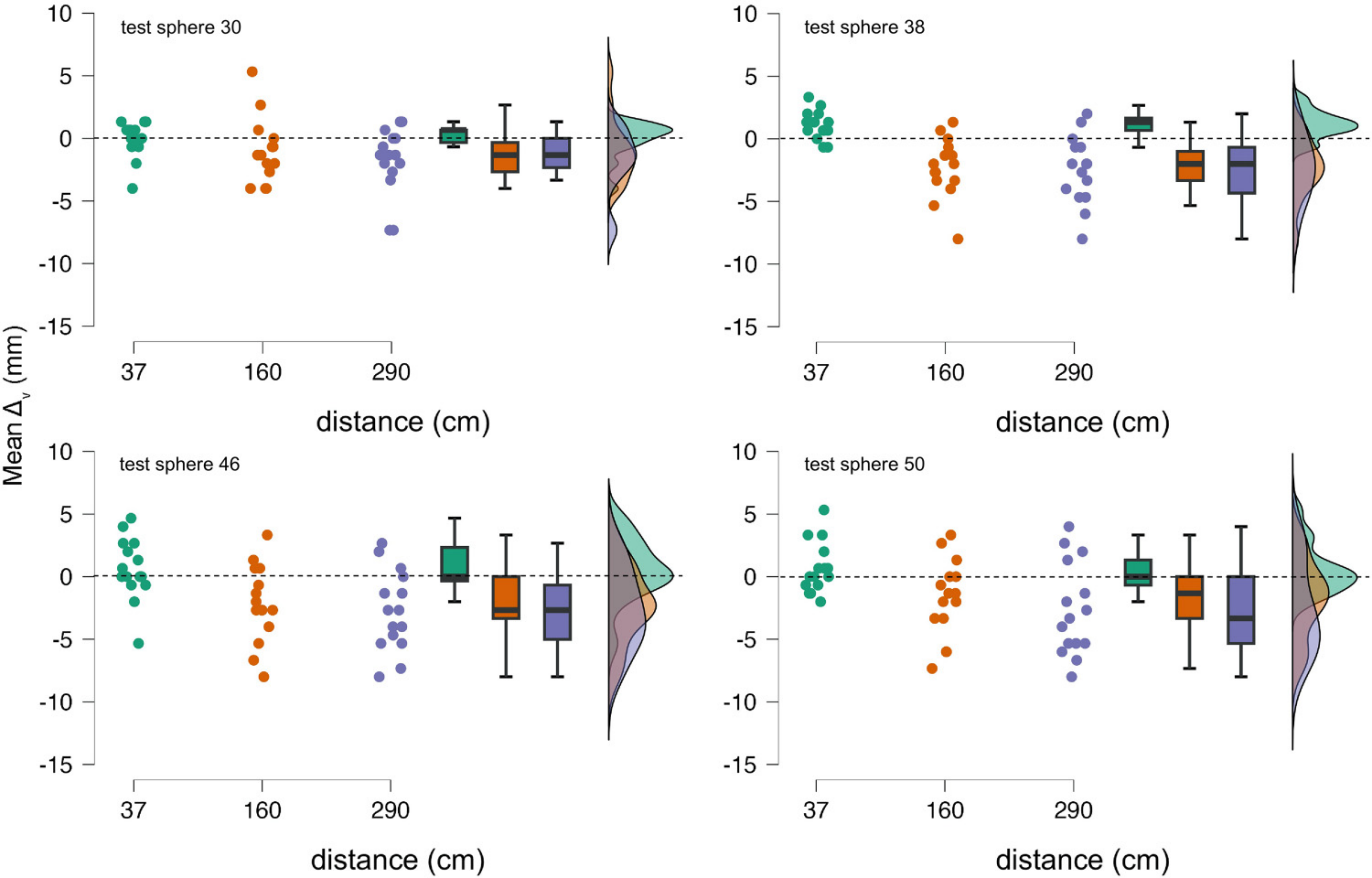
Experiment 2: data distributions for the four test spheres as a function of visual scale distance.

The most significant findings in Exp 2 are that: 1) when the visual scale is placed at a peripersonal distance (37 cm, i.e., within arm reach), participants’ matchings are roughly equivalent to the actual size of the test stimuli; 2) however, *Δ*_v_ values decrease with the visual scale set at extrapersonal distances (160 and 290 cm); in other words, test spheres are matched with smaller spheres on the visual scale with respect to their actual scale size. This result suggests that the sizes of the spheres in the visual scale are somewhat overestimated at such distances. In fact, given that the distance between participants and test stimuli is kept constant, yet these are matched with smaller spheres than their actual match when the scale is set at 160 and 290 cm from participants, it follows that the apparent sizes of the spheres in the scale increase with distance. It also important to underscore that mean *Δ*_v_ values for same scale distances are radically smaller than the mean *Δ* values found in Experiment 1. It is however important to underscore that as scale distance increases, participants’ mean matches are more scattered for both 160 and 290 cm distances (Figure 5).

## General Discussion

Two experiments were conducted to explore the ‘See What You Feel’ method, enhancing our understanding of perceptual mechanism across sensory modalities. The originality of this study lies in uncovering how the distance between participants and a visual scale influences a haptic-to-visual matching task.

Experiment 1 focused on the underestimation effect previously found in related research (Daneyko et al., 2020; Frisco et al., 2023), examining its consistency across different visual scale distances with the crossmodal (haptic-to-visual) matching task. This effort not only confirmed the persistent underestimation effect (handheld test spheres are matched with smaller spheres on the visual scale) across all tested distances, but also revealed that precision deteriorates with increasing distance, leading to more random matching outcomes. This highlights the complex dynamics in sensory integration and underscores the significance of exploring how scale distance – and, in more general terms, the distance of comparison stimuli – affects perceptual judgements. With the term ‘precision’ we do not refer to the size of *Δ*, which is the parameter that allows to determine whether a match is an under- or overestimation with respect to the actual size of a test sphere; we instead refer to the distribution of *Δ* values, that is to the density of the data cluster. Our findings align with results obtained in a relatively recent study on visuomotor processing by Bozzacchi and Domini (2015), where a notable effect of object distance on grip aperture was reported. In their study, grip aperture consistently decreased as object distance increased. Collectively, our findings suggest that both action (i.e., grasp) and perception may rely on similar visual information, underscoring the complex interplay between vision and haptic.

Experiment 2 shifted to a visually only matching task to isolate and examine the effect of scale distance without the complexities of crossmodal matching. Unlike Experiment 1, results for the peripersonal distance (37 cm) do not show an underestimation effect, suggesting that such effect appears to be the trademark of the SWYF method, therefore possibly reflecting a general transduction mechanism of tactile to visual information that characterises this crossmodal matching method.

Notably, Experiment 2 revealed that the spheres on a visual scale appear larger when placed at extrapersonal distances – a novel finding, as such an overestimation effect at relatively short distances has never been previously reported. However, the enlargement effect of the visual scale that we found comes at the cost of minor agreement among participants’ mean matches. This is an aspect that requires to be further investigated, also in view of the fact that our findings are not in line with those reported by Kavšek and Granrud (2012) at their viewing distance of 610 cm, which, however, exceeds our maximum viewing distance. In their experimental setup, test stimuli were placed at 200 cm from participants, that is, at a greater distance than in ours (32 cm).

A comparison between the results of experiments 1 and 2 with respect to scale distances set at 160 and 290 cm allow to speculate that there is an interaction between the haptic and visual domains in the size estimations of the test spheres in the SWYF method, with a general underestimation of the test spheres in the haptic domain and what appears to be an overestimation of the spheres composing the scale in the visual domain. It is challenging to determine whether this interaction affects matching results in a linear fashion because, as Figures 3 and 5 show, an increase in scale distance is accompanied by an overall increase in matching variability, which negatively affects the precision of the matching method.

With respect to the results of the visual-to visual matching task (Exp. 2), while size constancy theory typically predicts that the perception of object sizes should remain stable (Helmholtz, 1866; for a review of the size constancy issue also in relation to possible cortical underpinnings see Sperandio & Chouinard, 2015), numerous studies have reported a failure of size constancy at varying viewing distances (e.g., Granrud et al., 2003). Our findings support this by showing that size constancy is not observed with increasing distance between the scale and participants, in both the crossmodal matching context (Exp 1) and the visual-to-visual matching task (Exp 2). While results referring to experiment 1 are new and more experiments are indeed needed before speculating about the underpinning causes of the underestimations in relation to the visual component of the crossmodal matching task, experiment 2 shows that an overestimation of object size occurs already at a distance as short as 160 cm, an observation that is new in the field.

To summarise, results from the two experiments are the following:

1. In line with previous literature (Daneyko et al., 2020; Frisco et al., 2023; Héroux et al., 2024), the SWYF haptic-to-visual matching paradigm shows constant underestimation of test stimuli with respect to their actual size.
2. Results from exp. 2 support the claim that the underestimation effect appears to be a trademark of haptic-to-visual matching paradigms.
3. We confirm an effect of distance on the crossmodal matching paradigm.
4. The effect of distance in the crossmodal paradigm relates to an increment in uncertainty that appears to grow with the distance of the visual matching scale from the observer.
5. Results from exp. 2 also suggest that as the distance of the visual scale increases, its perceived size also grows, leading participants to match test spheres with smaller ones in a visual-to-visual matching task.
6. The size overestimation of objects has been reported for distances greater than 6 m; with exp. 2 we show that it occurs even at closer “out of reach” distances; hence there is a need for further investigations on the effect of scale distance in visual-to-visual matching paradigms.

In conclusion, this research extends our previous work by validating the SWYF method's utility in non-illusory contexts, demonstrating its reliability and precision as a general crossmodal matching tool when applied at ‘arm-reach’ distances. This method not only provides valuable insights into crossmodal sensory perception but also proves its effectiveness under conditions that ensure minimal cognitive strain and easy implementation. It is an excellent tool for accurately measuring haptic size perception, that could be especially beneficial in fields such as rehabilitation, virtual reality and ergonomic design. This research confirms the SWYF paradigm's potential for practical applications, fostering the way for its use in research on multisensory integration and feedback mechanisms.

## Notes

1. The Uznadze haptic size-contrast illusion (Uznadze, 1966) is an aftereffect obtained by simultaneously adapting both hands to haptic stimuli, such as spheres, which however differ in size. After the adaptation phase, two identical test stimuli, usually equal in shape to the adaptation ones but with size somewhat in between those of the two adapting stimuli, replace the latter. Participants report that the test stimulus clenched in the hand adapted to the small adapting stimulus as bigger than the physically equal test stimulus clenched in the hand of the big adapting stimulus.

2. The problem with haptically evaluating the size of a haptic illusion induced by an adaptation process is that the body part needed for the size evaluation (in our case a hand) would be affected by the adaptation process; hence the measurement obtained may not reflect the actual perceived size. For example, consider a scenario where one hand is adapted to a relatively small sphere, and the other to a relatively large sphere (the typical case for inducing the Uznadze haptic illusion). When the two adapting spheres are replaced by two test spheres that are physically identical to each other, the hand adapted to the small sphere will feel the test sphere as bigger than the one grasped in the other hand that was adapted to the large sphere. If the measurement task required the participant to touch a series of spheres to determine the size of the test sphere held previously there are two challenges: 1) the hand is likely still under the influence of the adaptation; 2) the participant must recall the size of the test sphere because they are no longer holding it.
